# Improving case detection of tuberculosis in hospitalised Kenyan children—employing the behaviour change wheel to aid intervention design and implementation

**DOI:** 10.1186/s13012-020-01061-4

**Published:** 2020-11-25

**Authors:** Jacquie Narotso Oliwa, Jacinta Nzinga, Enos Masini, Michaël Boele van Hensbroek, Caroline Jones, Mike English, Anja van’t Hoog

**Affiliations:** 1 KEMRI-Wellcome Trust Research Programme, Nairobi, Kenya; 2 Department of Paediatrics and Child Health, School of Medicine, University of Nairobi, Nairobi, Kenya; 3 The Academic Medical Centre, Department of Global Health, Faculty of Medicine, University of Amsterdam, Amsterdam, The Netherlands; 4 The Global Fund, Geneva, Switzerland; 5 Amsterdam Institute for Global Health and Development, Amsterdam, The Netherlands; 6 Nuffield Department of Medicine, Centre for Tropical Medicine and Global Health, Oxford University, Oxford, UK

**Keywords:** Tuberculosis, Child, Case detection, Diagnostics, Hospitalised, Implementation, Intervention, Behaviour change

## Abstract

**Background:**

The true burden of tuberculosis in children remains unknown, but approximately 65% go undetected each year. Guidelines for tuberculosis clinical decision-making are in place in Kenya, and the National Tuberculosis programme conducts several trainings on them yearly. By 2018, there were 183 GeneXpert® machines in Kenyan public hospitals. Despite these efforts, diagnostic tests are underused and there is observed under detection of tuberculosis in children. We describe the process of designing a contextually appropriate, theory-informed intervention to improve case detection of TB in children and implementation guided by the Behaviour Change Wheel.

**Methods:**

We used an iterative process, going back and forth from quantitative and qualitative empiric data to reviewing literature, and applying the Behaviour Change Wheel guide. The key questions reflected on included (i) what is the problem we are trying to solve; (ii) what behaviours are we trying to change and in what way; (iii) what will it take to bring about desired change; (iv) what types of interventions are likely to bring about desired change; (v) what should be the specific intervention content and how should this be implemented?

**Results:**

The following behaviour change intervention functions were identified as follows: (i) training: imparting practical skills; (ii) modelling: providing an example for people to aspire/imitate; (iii) persuasion: using communication to induce positive or negative feelings or stimulate action; (iv) environmental restructuring: changing the physical or social context; and (v) education: increasing knowledge or understanding. The process resulted in a multi-faceted intervention package composed of redesigning of child tuberculosis training; careful selection of champions; use of audit and feedback linked to group problem solving; and workflow restructuring with role specification.

**Conclusion:**

The intervention components were selected for their effectiveness (from literature), affordability, acceptability, and practicability and designed so that TB programme officers and hospital managers can be supported to implement them with relative ease, alongside their daily duties. This work contributes to the field of implementation science by utilising clear definitions and descriptions of underlying mechanisms of interventions that will guide others to do likewise in their settings for similar problems.

**Supplementary Information:**

The online version contains supplementary material available at 10.1186/s13012-020-01061-4.

Contributions to literature

- Implementation studies have been criticised for lack of conceptual/theoretical clarity and inconsistent use of terminologies, making them difficult to replicate.
- We used the Expert Recommendations for Implementing Change (ERIC) taxonomy to ensure consistent language in our intervention design, which adds to the body of work that can be comparable in future reviews of implementation studies.
- We also used theory guided by the Behaviour Change Wheel to propose how change should occur and to describe the underlying mechanisms of change that will guide others proposing to do likewise in their settings. This was particularly helpful for the complex longstanding problem of diagnosing tuberculosis in children, for which using behavioural approaches provided a range of insights that guided development of an intervention.
- We demonstrated the use of theory to describe intervention components and to explain how they will achieve their effects that will enhance transferability of our findings to other settings that grapple with same issues as we do in diagnosing TB in children.
- We demonstrated the importance of the use of local empiric data to ensure the intervention is designed for the context: any existing efforts for paediatric TB in Kenya have been adaptations of WHO recommendations by local experts.

## Background

Tuberculosis (TB) is a leading cause of morbidity and mortality in children. According to the World Health Organisation (WHO), there were approximately 1.12 million incident child TB cases in 2018 and 205,000 deaths [1]. The true burden remains unknown due to challenges in diagnosis, but it is estimated that up to 65% of TB cases in children < 5 years go undetected each year [2–4]. In Kenya, 75% of TB cases identified in a recent population-based survey had visited health facilities with suggestive symptoms but were never diagnosed [5]. Our work has shown that failure to detect tuberculosis in children who are already admitted in hospital represents a missed opportunity [6]. Guidelines for TB clinical decision-making are in place in Kenya, adapted from global resources, and the National TB programme (NTP) conducts training on those guidelines every year, as part of its strategic plan [7–10]. WHO recommends the use of Xpert® MTB/RIF (Xpert®) as a first-line TB diagnostic test and by 2018 there were 183 machines in Kenya in public hospitals across the country [10]. Despite these efforts by the NTP of training and making machines available, underuse of TB diagnostic tests in Kenya is quite high [6, 11].

Research on factors that are likely to enhance or constrain the uptake of new evidence or tools into clinical practice is becoming more common [12–18]. Implementation science looks at the best approaches to move research into practice to improve the quality and effectiveness of health services, and focuses a lot on changing healthcare professional and organisational behaviour [19]. Implementation studies have however been criticised for lack of conceptual/theoretical clarity and inconsistent use of terminologies, making them difficult to replicate [20, 21]. Theory is important to guide the process of implementation, to explain what influences implementation outcomes and evaluate implementation [22]. The linkage of theory with intervention design is recommended by the Medical Research Council (MRC) guidance on the development and evaluation of complex interventions [23, 24]. Systematic use of theory aids delivery of evidence-informed strategies adapted to the local context [21, 25, 26]. However, programmatic interventions in low-resource settings are still often only input focused, for example, the major focus of the Kenya TB programme has been increasing provision of GeneXpert® machines, training more staff and distributing more guidelines [10].

We describe the process we undertook to design a contextually appropriate and theory-informed intervention to improve case detection of TB in children in Kenyan hospitals guided by the Behaviour Change Wheel (BCW) [27]. We chose the BCW, recognising that individual and collective behaviour change is key to implementing new practices and to improve health outcomes [22, 28–30]. One of the strengths of the BCW is that it naturally incorporates context, which is key to effective design and implementation of interventions [29]. The BCW is anchored on the Theoretical Domains Framework (TDF), an integrative framework of 33 psychological theories related to behaviour change, synthesised in a way that enables systematic assessment of implementation issues to inform intervention design, and is explained further in subsequent sections [30]. We also used the Expert Recommendations for Implementing Change (ERIC) taxonomy to ensure consistent language [20]. This work thus aimed to develop a clear starting perspective to design an intervention that could feasibly be adopted, evaluated and scaled up by the National TB Programme (NTP). We used information from our empiric data [6, 11, 31, 32], literature and discussions with key stakeholders to gain a deep understanding of context to support choice of intervention strategies. Whilst focused on Kenya, we hope this work will be of value to others in similar contexts working to improve effectiveness of TB care for children.

## Methods

### Setting

**Fig. 1:**
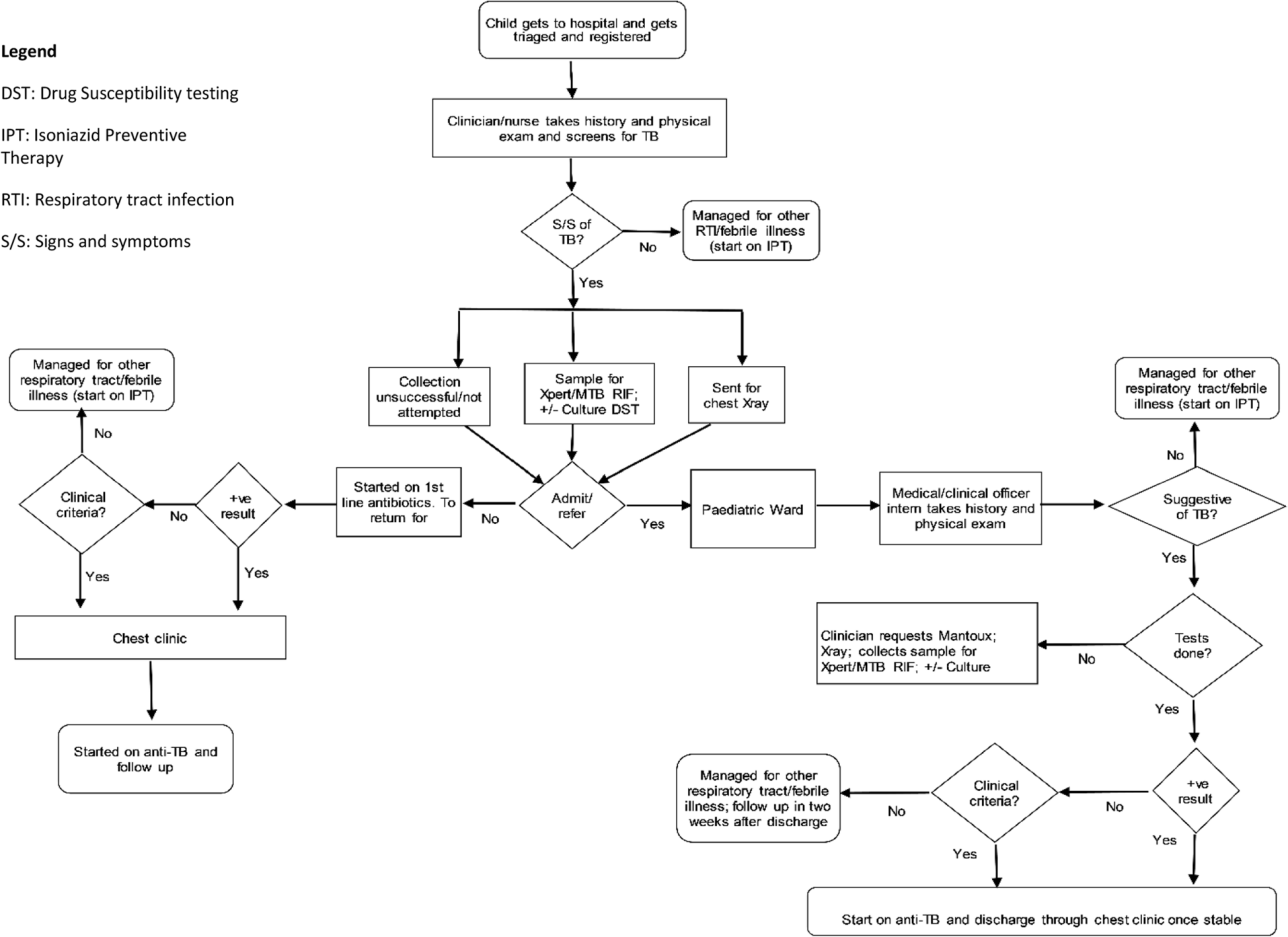
Process map showing patient flow of a probable TB case through typical county hospital

Kenya has a young population, 73% of its approximately 48 million inhabitants are below 30 years of age. It is classed as a low-middle-income country with a gross national income (GNI) per capita of $1600 but 36.1% of the population lives below the poverty line [33]. Kenya is one of the 30 TB high-burden countries, with a prevalence of 426 per 100,000 and case detection rate of 64%, with children representing 9-10% of the notified cases [34]. Most Kenyans receive inpatient hospital services from public health facilities. These are classified in three tiers (levels 4-6) with lower tiers (levels 1-3) offering community and primary care. Sub-county hospitals (level 4) may be run by a clinical officer or a medical officer or a specialist medical practitioner. County hospitals (level 5) may be run by a medical officer or a specialist. National referral hospitals (level 6) are run by fully qualified specialist medical practitioners. The focus of the work that has led to this paper is the management of children hospitalised in Kenyan county and sub-county hospitals, all of which have at least one GeneXpert® machine, or access via specimen referral. The process map derived from previous work [31] and replicated in Fig. 1 shows how children with possible tuberculosis are processed within these hospitals, and illustrates the local context. Our earlier work helped to identify bottlenecks within this context and contributing factors to these bottlenecks are the starting points for the intervention design described in this paper.

### Using the Behaviour Change Wheel to guide intervention design

**Fig. 2:**
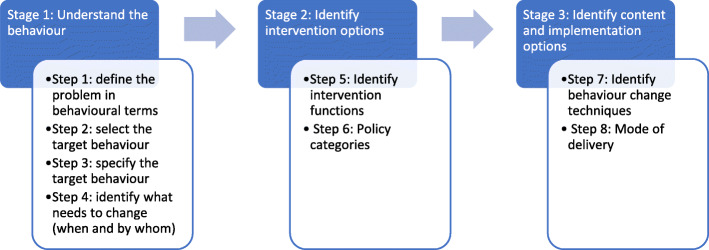
Steps in intervention design [27]

The Behaviour Change Wheel (BCW) is a framework that supports systematic development of interventions [27, 29]. It is designed to facilitate systematic, evidence-based progression from behavioural analysis of a problem to intervention design employing behaviour change theory to bring about desired change in three stages as shown in Fig. 2.

**Fig. 3:**
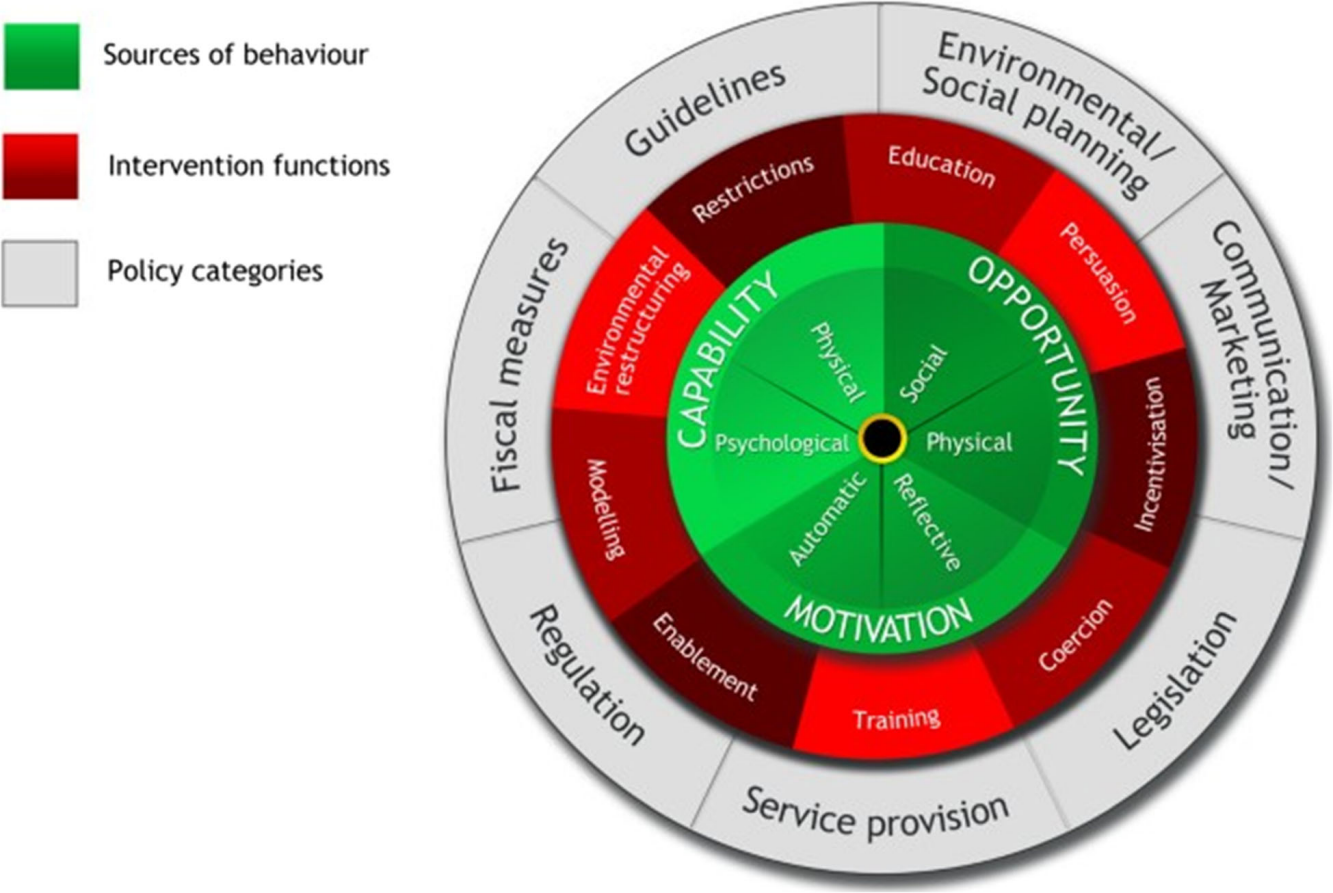
The Behaviour Change Wheel [27, 29]

The BCW is made up of three layers as shown in Fig. 3, and fully described in the Guide to Designing Interventions and accompanying article [27, 29]. The core is formed by the *Capability, Opportunity* and *Motivation* Behavioural (COM-B) theoretical model. *Capability* is defined as one’s psychological capacity (knowledge, memory) and physical capacity (strength, skills, stamina) to engage in an activity/behaviour. *Opportunity* represents factors that lie outside the individual that affect one’s capacity to perform, and include time, physical environment, interpersonal influences, social cues and cultural norms. *Motivation* represents internal factors (brain processes) that allow one to employ *capability* and *opportunity* to perform a behaviour, and include wants, needs, impulses, habits, beliefs, intentions and choices [29]. COM-B model thus explains conditions internal to individuals and within their social and physical environment necessary for them to enact a desired behaviour, which in our case is to correctly diagnose TB in children [29]. COM-B is the starting point used by the Behaviour Change Wheel for understanding behaviour in the context in which it occurs. Surrounding the core are interventions which mainly target individuals, e.g. education, coercion or act at policy level, e.g. guidelines and fiscal measures.

Each of the COM-B components maps onto the Theoretical Domains Framework (TDF)—a synthesis of 33 theories and 84 theoretical constructs of behaviour change organised into 14 domains [21]. The domains thought to be relevant to health workers’ change in behaviour include knowledge; skills; memory, attention and decision processes; behavioural regulation; social/professional role and identity; beliefs about capabilities; optimism; beliefs about consequences; intentions; goals; reinforcement; emotion; environmental context and resources and social influences [28, 35]. The TDF therefore provides a theoretical basis for implementation research, to aid understanding of which interventions are likely to work and why. Behaviour Change Techniques (BCTs) are the active, observable and replicable components of an intervention designed to change behaviour, i.e. the proposed mechanism of change and commonly used examples include problem solving, feedback on outcomes, instruction on how to perform a behaviour, restructuring the physical environment, prompts and cues etc. [27]. COM-B/BCW has been used successfully for behavioural analysis and to design interventions in both health and non-health-related fields [26, 36–55], but to our knowledge, has been used in only one study of TB on contact tracing in a low-resource setting, to identify barriers and facilitators and to tailor interventions to improve contact investigation in Kampala [26].

### Data collection (stage 1: Understanding the behaviour)

We used a mixed-methods strategy (Additional file 1) to collect empirical data to identify challenges in case detection of TB in children to enable behavioural analysis. For the quantitative arm, we analysed national TB programme data as well as data from children admitted to 13 county hospitals in Kenya to describe the burden of childhood TB and diagnostic practices and these have been reported elsewhere [6, 11]. Results show at national level, there is under detection of TB in children and underuse of available TB diagnostic tests. At hospital level, we found more than half of all paediatric admissions in Kenyan county hospitals had signs and symptoms suggestive of TB, but in most, TB was not considered as a differential diagnosis. Only 1% of these children meeting criteria for diagnostic testing had an Xpert® MTB/RIF assay performed, which was available in all the hospitals.

In the qualitative arm, to understand the challenges in recognising and testing for TB in admitted children we analysed data from (i) semi-structured interviews, small-group discussions and key informant interviews with front-line health workers and mid-level managers; (ii) observations of TB trainings, sensitisation meetings, policy meetings and hospital practices and (iii) desk review of guidelines, job aides and policy documents, which have been reported elsewhere [31]. We used the COM-B framework to interpret emerging themes. At individual level, we found that knowledge, skill, competence and experience, as well as beliefs and fears impacted on *capability* (physical and psychological) as well as *motivation* (reflective) to think of TB as a differential diagnosis in children and use diagnostic tests. Hospital level influences included hospital norms, processes and patient flows and resources which affected how individual health workers attempted to diagnose TB in children by impacting on their *capability* (physical and psychological), *motivation* (reflective and automatic) and *opportunity* (physical and social). At the wider system level, community practices and beliefs, and implementation of TB programme directives impacted some of the decisions that health workers made through *capability* (psychological), *motivation* (reflective and automatic) and *opportunity* (physical).

### Behavioural analysis and intervention design: Identifying intervention options, content and implementation options (stage 2 and 3)

As a study team, we used an iterative brainstorming process over several meetings during the study period (an average of weekly for the lead investigator and research assistant, and monthly for the larger study team, with increased frequency during study onset and analysis). During discussions at these meetings, we went back and forth from the quantitative and qualitative empiric data to reviewing literature, and applying the BCW guide [27]. The key questions reflected on included (i) what is the problem we are trying to solve; (ii) what behaviours are we trying to change and in what way; (iii) what will it take to bring about desired change; (iv) what types of interventions are likely to bring about desired change; (v) what should be the specific intervention content and how should this be implemented?

The empiric data helped identify gaps in case detection of TB in children and use of diagnostic tests in Kenya. We used COM-B and TDF to map out these gaps in behavioural terms, i.e. to identify and specify what actions need to change and by who to address the gaps. Behavioural analysis involves the consideration of conditions internal to individuals and in their social and physical environment that need to be in place for a particular target to be achieved [29].

**Table:** 

Panel illustrating a worked example of behavioural analysis ***What is the problem from empiric data****: gaps in the evaluation of children for TB.* ***What behaviour needs to change:*** *better documentation of signs and symptoms suggestive of TB in children* ***By who****: all clinicians seeing sick children.* ***When:*** *at each patient encounter* Examples of some relevant COM-B elements, TDF constructs, intervention functions, policy functions, behaviour change techniques and mode of delivery (as per BCW guide steps) ***i) Capability****: clinicians need to know the importance of correctly identifying TB in children, and the skills to identify the key signs and symptoms);* ***TDF construct****: Knowledge- awareness of the steps in diagnosing TB in children* ***Intervention function:*** *Training to impart skills; modelling to provide a credible example* ***Policy function:*** *Guidelines - to ensure availability and access to child TB protocols* ***Behaviour change techniques:*** *Instruction on how to perform the behaviour (Training); Demonstration of the desired behaviour (Modelling)* ***Mode of delivery:*** *Face-face to individuals & groups (training); print media (guidelines)* ***ii) Opportunity:*** *the time to do proper assessment, structured forms that prompt documentation, culture of providing quality care;* ***TDF construct:*** *Social influences: group conformity to good clinical practices* ***Intervention function****: environmental restructuring to ensure availability of structured forms; Modelling- providing credible examples* ***Policy function:*** *Regulation (establishing principles of best practice)* ***Behaviour change techniques:*** *adding objects to the environment (structured forms); demonstration of the behaviour (champions)* ***Mode of delivery:*** *Face-face to individuals and groups* ***iii) Motivation:*** *belief that failure to correctly evaluate children could lead to missed diagnosis and death* ***TDF construct:*** *Beliefs about consequences* ***Intervention function:*** *Persuasion- using audit and feedback of missed diagnosis, adverse outcomes* ***Policy:*** *Regulation- requirement of regular audits* ***Behaviour change techniques:*** *Feedback on behaviour* ***Mode of delivery:*** *Face-face to individuals and groups*	

We used the BCW to link the gaps to evidence-based intervention functions like education, persuasion, environmental restructuring and these were in turn linked to policy categories. The panel illustrates a worked example of this process and Additional files 2, 3, 4 have lengthier descriptions of the steps we followed during behavioural analysis as illustrated in Figs. 2 and 3 from the BCW guide [27].

We used the experience of the research team including implementation scientists, epidemiologists, social scientists, clinicians and clinician educators, together with feedback from clinical colleagues to select potential interventions (Table 1). We focused on those behaviour change techniques and modes of delivery that would yield results at low cost and that could feasibly be taken up by the National TB Programme.

**Table 1:** Linking gaps in empiric data for behavioural analysis to intervention design (stages 1 and 2)

Summary of gaps identified in empiric data from our previous studies	COM-B	TDF constructs linked to COM-B	Relevance of the theoretical domain	Proposed intervention function from the BCW guide [36]
Under-detection of TB in children, 60-70% thought to be missed (QUAN) Nearly 60% of all paediatric admissions met guideline criteria for suspected TB but < 3% got a diagnosis (QUAN)	Capability-psychological	Knowledge Behavioural regulation	Awareness of steps in diagnosing TB in children; of the available tests. Do they know what they should do and when and why? Self-monitoring; how to break a habit e.g. missed diagnosis. Anything in place to prompt them to make a diagnosis and to self-monitor?	Training: Imparting skills on how to correctly diagnose TB in children Modelling: Providing an example for people to aspire/imitate, e.g. via champions/clinical leaders Persuasion: Using communication to stimulate action, e.g. via audit and feedback
Some reported that they did consider a TB differential diagnosis but sometimes forgot to document (QUAL) Some reported they do tests but forgot to document (QUAL)	Capability-psychological	Memory attention and decision processes Behavioural regulation	Ability to retain information, to consistently remember to document what is done Self-monitoring; how to break a habit, e.g. failure to document. Anything in place to prompt them to always document?	Environmental restructuring: Changing the physical context, e.g. availability of record forms for better documentation, job aides Persuasion: Using communication to induce positive or negative feelings or stimulate action, e.g. via audit & feedback; shared goals with peers
Some health workers fear/are reluctant to make a diagnosis of TB in children sometimes due to stigma in caregivers of TB-HIV association (QUAL)	Capability-psychological Motivation-automatic	Knowledge Reinforcement Emotion	Awareness of steps in diagnosing TB in children; of the available tests. Do they know what they should do and when and why? Anything to motivate or demotivate them? Does it evoke an emotional response, e.g. some got uncomfortable when babies cried during specimen collection; some were reprimanded harshly by caregivers	Education: Increasing knowledge or understanding of TB in children Persuasion: Building communication skills to better counsel families Modelling: by the champions to demonstrate how best to de-stigmatise
Underutilisation of TB diagnostic tests, 1% get Xpert done (QUAN) Health workers generally seem to have a challenge in collecting specimen for children (QUAL)	Capability-psychological Capability-physical Motivation-reflective Motivation-automatic	Knowledge Physical skills Beliefs about capability Reinforcement	Awareness of steps in diagnosing TB in children; of the available tests. Do they know what they should do, when and why? Are they physically able/proficient in diagnosing TB; collecting specimen; using diagnostic tests? Acquired through practice Are they confident diagnosing TB in children; collecting specimen? How difficult or easy? Increasing likelihood of TB tests being used appropriately	Training: Imparting skills to use available diagnostic tests and specimen collection Modelling: Champions/clinical leaders demonstrating correct procedures Environmental restructuring: identifying who is responsible for ensuring TB tests get done; job aides to serve as reminders of procedures
Health workers report consistently negative Xpert test results (QUAL)	Capability-psychological Motivation-reflective	Knowledge Beliefs about consequences	Do they know how to respond to negative test results? How and when to make a clinical diagnosis? Do they believe doing it or not makes a difference?	Education: increasing understanding on making a clinical diagnosis and the epidemiology and natural course of TB in children Persuasion: communication to pass on the value of TB tests
Some facilities had good teamwork and mentorship that helped model the correct way to diagnose TB in children (QUAL)	Opportunity-social Motivation-reflective	Social/professional role and identity Optimism	Do they think it is part of their job, e.g. to collect specimen (senior doctors struggled) Do they think it’s something that can be done? How confident are they of this?	Modelling and social environment restructuring: Providing an example for people to aspire/imitate and encouraging teamwork Persuasion: communication to pass on the value of diagnosing TB in children
Most facilities had long and unclear processes that contributed to TB being missed in children (QUAL) Some reported frequent stock-outs of some reagents and XPert cartridges (QUAL)	Opportunity-physical	Environmental context and resources	Organisational processes and patient flows; resources like job aides, PPE, reagents. Aspects of the environment that influence whether or not they diagnose TB in children	Environmental restructuring: Changing the physical context to ensure better work flows and availability of equipment, reagents
Lack of skilled human resource to interpret some test results like chest X-rays (QUAL)	Opportunity-physical Capability-psychological	Environmental context and resources Knowledge	Aspects of the environment that influence whether or not they diagnose TB in children Awareness of steps in diagnosing TB in children; of the available tests. How to make a clinical diagnosis?	Environmental restructuring: e.g. job aides to guide clinical diagnosis; remote decision-support for X-ray interpretation Training: Imparting skills of reading X-rays looking for TB-specific features; making a clinical diagnosis
Some policies and directives including selection of participants for training disadvantaged front-line health workers (QUAL)	Opportunity-physical Motivation-automatic	Environmental context and resources Reinforcement	Aspects of the environment that influence whether or not they diagnose TB in children Anything to motivate or demotivate? (lack of training was a demotivator)	Education: increasing policy makers’ understanding of the need of rethinking how TB training is done Persuasion: Using communication to stimulate action, e.g. feedback to policy makers on the impact of training
TB programme policy of doing quarterly audits and supervisory visits helped (QUAL)	Motivation-reflective	Intentions Goals	Feedback to enable health workers to make a conscious decision to improve case detection Visualise what they want to achieve	Persuasion: Using communication to stimulate action, e.g. via audit & feedback

Using information gathered from our empirical data, literature on interventions likely to be successful, [56, 57], our understanding of the context and taking the perspective of what would be feasible for hospital managers and NTP officers to implement, we came up with a list of possible interventions to address the gaps in diagnosing TB in children. We then further selected options linked to the predicted mechanism of change according to the TDF constructs and used the APEASE criteria^1^ to rationalise in terms of affordability, practicability, effectiveness, acceptability, safety and equity [27]. We presented findings to key paediatric TB stakeholders (including NTP officials, developmental partners, paediatricians and academic staff). We had informal discussions during technical working group meetings (there were two during the study period) to gain their perspectives on what could work, after considering our local context.

Table 1 summarises the process of linking the gaps in empiric data through the major Behaviour Change Wheel design steps. The first column gives a summary of the key findings from our previous studies, and these were linked to the various COM-B elements and TDF constructs, and proposed intervention functions from the BCW guide.

Relevant aspects of The Standard for Reporting Implementation Studies (STaRI) tool [58] were used to help ensure key elements needed when developing and evaluating implementation strategies have been covered to enhance adoption and sustainability (see Additional file 5).

## Results

From the behavioural analysis, the following behaviour change intervention functions were identified: (i) training: imparting practical skills conducted by the National TB Programme (NTP); (ii) modelling: providing an example for people to aspire/imitate by champions/clinical leaders; (iii) persuasion: using communication to induce positive or negative feelings or stimulate action via audit and feedback by the ward clinical leaders and/or TB programme staff; (iv) environmental restructuring: changing the physical or social context, e.g. availability of record forms for better documentation; and (v) education: increasing knowledge or understanding by the champions. From these, the following policy categories were identified: (i) guidelines: ensuring availability and access to child TB diagnostic protocols by the NTP; (ii) regulation: establishing principles of best practice by the NTP; and (iii) communication/marketing: conducting mass media campaigns to educate the public on TB by the NTP and mass marketing to target health workers on the need to scale up TB testing.

**Fig. 4:**
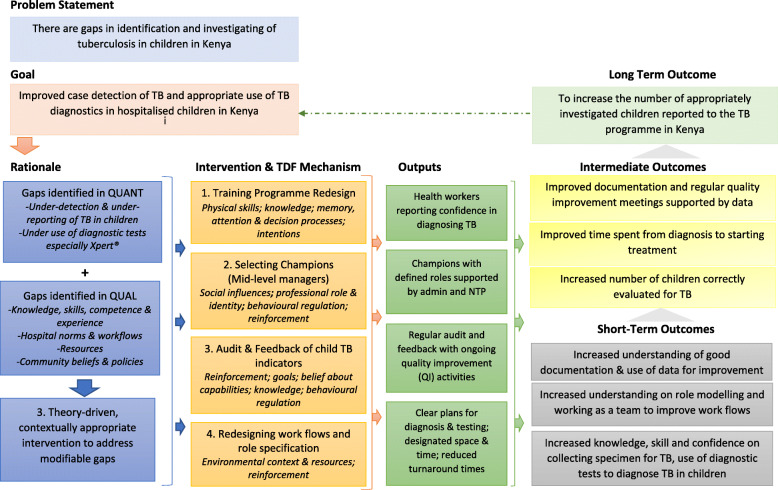
Theory of change for a multi-faceted intervention to improve case detection of tuberculosis in children in Kenya

From discussions with the various child TB stakeholders, a multi-faceted intervention package composed of redesigning of training to focus on practical skills, selection of champions, better use of audit and feedback and workflow restructuring was proposed. Table 2 summarises the process that was followed in linking our intervention package with theory. The intervention components are defined using ERIC taxonomy, after considering the BCW guide intervention functions. The logic model (Fig. 4) conceptualises the theory of change of how the intervention package might work.

**Table 2:** Linking interventions with behaviour change techniques and mode of delivery

Intervention (as defined by ERIC taxonomy)	Target behaviour	Behaviour change technique	Mode of delivery	Major gaps using APEASE criteria A-Affordability P-Practicability E-Effectiveness A-Acceptability SE-Side Effects E-Equity
Training programme redesign	On-job training HCWs in child TB (specimen collection, interpreting CXRs)	*Instruction on how to perform the behaviour* *Demonstration of the behaviour*	Face-face to individuals and groups Print media (guidelines)	Low practicability: needs skilled staff to train and time off busy schedules
Purposeful selection of champions	Providing clinical leadership, mentorship and supervision Building teamwork to ensure best practices	*Demonstration of the behaviour* *Credible source* *Social support* *Goal setting* *Feedback on the behaviour*	Face-face to individuals and groups	Low practicability: low where staff are few and stretched and none willing to take up role
Audit and feedback	Encourage better documentation of history and physical signs and symptoms suggestive of TB Encourage better documentation of tests ordered and date done Encourage better documentation of samples collected, when and test results	*Adding objects* (*record forms*) *to the environment* *Feedback on the behaviour* *Prompts/cues*	Face-face to individuals and groups Individually accessed computer-generated reports	Low acceptability: may resist if not part of their culture Practicability: low where staff are few and stretched
Workflow restructuring	Reorganising patient flow and processes Ensuring samples get to the lab on time Ensuring results get back to each patients’ file and gets reviewed by clinician	*Restructuring of the physical and social environment* *Feedback on the behaviour* *Prompts and cues* *Demonstration of the behaviour*	Group	Low practicability and acceptability: may be low where staff are few and stretched
Resources	Ensuring availability of reagents, cartridges, specimen bottles, safety masks Ensuring availability and use of guidelines/job aides Providing personal protective equipment and encouraging consistent use	*Restructuring of the physical environment* *Adding objects to the environment* *Feedback on the behaviour* *Demonstration of the behaviour* *Prompts and cues*	Group Individual—in-charge: using reports	Low affordability: cost prohibitive Low acceptability: using masks Low effectiveness: of procurement Low availability: dependent on TB programme Low acceptability: low where people prefer to use their acumen

The subsequent section looks at each component in turn, elucidating selected BCW interventions functions using the definitions as per the Expert Recommendations for Implementing Change (ERIC) taxonomy [20], briefly reviewing available evidence for how each may impact health worker practice and how they would be delivered in our context.

### Redesigning of training

Training is defined as giving instruction and/or actual demonstration of the desired action and works to improve physical and psychological capabilities of health workers, and with time, their reflective and automatic motivation [20]. The theoretical constructs through which training work are physical skills: memory, attention and decision processes [27]. The child TB training has traditionally been didactic/classroom based, usually away from the providers’ facility (to remove interruptions from work) and NTP has trained hundreds of health workers in this way. Training is a key component of the NTP national strategic plan and receives a considerable budget every year [10]. Feedback from Kenyan health workers was that they felt they still lacked competence in specimen collection in children and how to interpret test results. There was also concern about the selection of participants for training-key frontline actors were often left out [31].

ERIC recommend that training should be made dynamic, i.e. vary the information delivery methods to cater to different learning styles and work contexts, and shape the training to be interactive [20]. The evidence however shows that training on its own has modest effects on health worker performance and propose that it should be combined with other strategies like supervision and group problem solving [57]. We recommend that child TB training be made more hands on, with skills being demonstrated and participants are given opportunities to practice under supervision until competence is attained. The mode of delivery should be both to individuals and groups, preferably at their workplaces, initially using video demonstrations and then with actual patients. Ongoing training in the form of continuous medical education/refresher sessions can be arranged ideally led by the champions. Training can be supplemented with educational outreach visits—having a trained person meet with providers in their practice settings to educate providers about TB in children with the intent of changing their practice. Redesign and distribution of printed material like guideline booklets and posters to remind health workers of the correct steps and procedures are an additional suggested mode of delivery of training as an intervention.

### Champions/local opinion leaders

Champions are usually local opinion leaders, individuals perceived as credible and trustworthy and disseminate and implement best evidence, for instance through informal one-to-one teaching [59]. According to ERIC, these are individuals who dedicate themselves to supporting, marketing and driving through an implementation, overcoming indifference or resistance that the intervention may provoke in an organisation [20]. They provide clinical leadership, mentorship and supervision through modelling/demonstrating the correct procedures and this should impact health workers’ reflective and automatic motivation positively—important in places where leadership is largely lacking [31]. The main theoretical construct through which champions work to improve health worker practices is through social influence and reinforcement. A recent Cochrane review found that local opinion leaders alone or in combination with other interventions probably improve health workers’ compliance with evidence-based practice but the effect on patient outcomes is uncertain [59]. Another review found that combining training and supervision had somewhat larger effects than use of either strategy alone [57]. We recommend selection of willing mid-level managers like paediatricians, senior medical or clinical officers and nurse managers in county hospitals to perform this champion role, together with the TB clinic teams. Our work found that paediatricians in particular are often left out of child TB trainings and policy decisions, and yet a final decision to start TB treatment in difficult to diagnose patients is often left to them [31]. The NTP now recognises them as opinion leaders and has had several sensitisation meetings to update them on the latest guidelines and engage them as partners in improving care. The champions should be supported with leadership training to enable them to perform their roles.

### Audit and feedback with group problem solving

Audit and feedback involves collecting and summarising clinical performance data over a specified time period and giving it to clinicians and administrators to monitor, evaluate and modify provider behaviour [20]. We found that the NTP regularly collect data from patients started on treatment but the hospital teams were not consumers of these data. The audit is done at the county level, but feedback is mainly given to the county TB coordinators and clinicians at the TB clinics, excluding those on the wards [31].

Audit and feedback has been widely used based on the belief that healthcare workers will be prompted to modify their practice when given feedback showing their behaviour is inconsistent with a desirable target [12]. Ivers et al. showed that audit and feedback generally leads to small but potentially important improvements in professional practice [12]. The effectiveness depends on baseline performance and how the feedback is provided. We propose that feedback from national level could be given by the TB county coordinators or by the champions to all the clinical teams on the quality of care given to children with possible TB. This then sets the stage for local audits and group problem solving led by the champions/clinical leads or TB coordinators. Audit and feedback will target health workers’ psychological capability and eventually their reflective and automatic motivation. The theoretical constructs through which audit and feedback work include reinforcement and behavioural regulation.

Group problem solving has been shown to have moderate to large effects on improving health worker practices [57]. According to ERIC, group problem solving could work through clinician implementation team meetings. Initiating these may require some coaching and they would require protected time to reflect on the implementation effort, share lessons learned and support one another’s learning [20]. These teams should ideally bring together representation from clinicians from the TB clinic, laboratory personnel, biomedical teams and clinicians in the wards and out-patient departments, as our work showed gaps in teamwork leading to bottlenecks in patient flows [31]. For feedback to work well, there needs to be credible data, and this requires good documentation as the initial step. Good documentation requires environmental restructuring to ensure consistent availability of structured record forms, laboratory forms and other records.

### Workflow restructuring

We observed several bottlenecks in patient flow and processes that were a hindrance to identifying potential TB patients in hospitals, as illustrated in the following vignette:

**Table:** 

Workflow vignette *An example is given of a child with possible TB in a busy outpatient department. The patient was sent to the laboratory with a request form for investigations, as the clinician was alone with long queues and had no designated space or time to collect specimens. The laboratory technician said it was not his job to collect samples and he was also alone, so the patient was sent to the ward to request the junior doctor to assist. Unfortunately, she was new in the ward and had never done specimen collection for TB in children and was busy with other procedures for the ward admissions and could not help. After spending the whole day in and out of various departments, the child and the caregiver were sent back where they started, only to find their clinician left for the day, and a new clinician had started a shift.*	

ERIC describe an intervention strategy of changing physical structure and equipment. This requires one to evaluate current configurations and adapt as needed the physical structure and/or equipment, e.g. changing the layout of a room, adding equipment to best accommodate the targeted innovation [20]. Reorganising patient flow and processes targets physical and social opportunity as well as reflective and automatic motivation and works through TDF constructs of reinforcement, knowledge and behavioural regulation. Workflow also encompasses social restructuring with a clear definition of roles and expectations, e.g. who should collect samples, where and when. We propose that workflow restructuring be done with the local clinical implementation teams, as part of earlier described group problem-solving activities, where they restructure and keep adapting until they reach the best local solutions. The use of process maps such as Fig. 1 can help with this. It is important to ensure holistic care of all patients, so that improved TB care for children is done in tandem with improving quality of care for all. Workflow restructuring has been shown to improve health worker practices as they are based on local problem analysis and generation of solutions. The health workers get empowered because they gain control over their own work [60].

### Implementation and evaluation

**Fig. 5:**
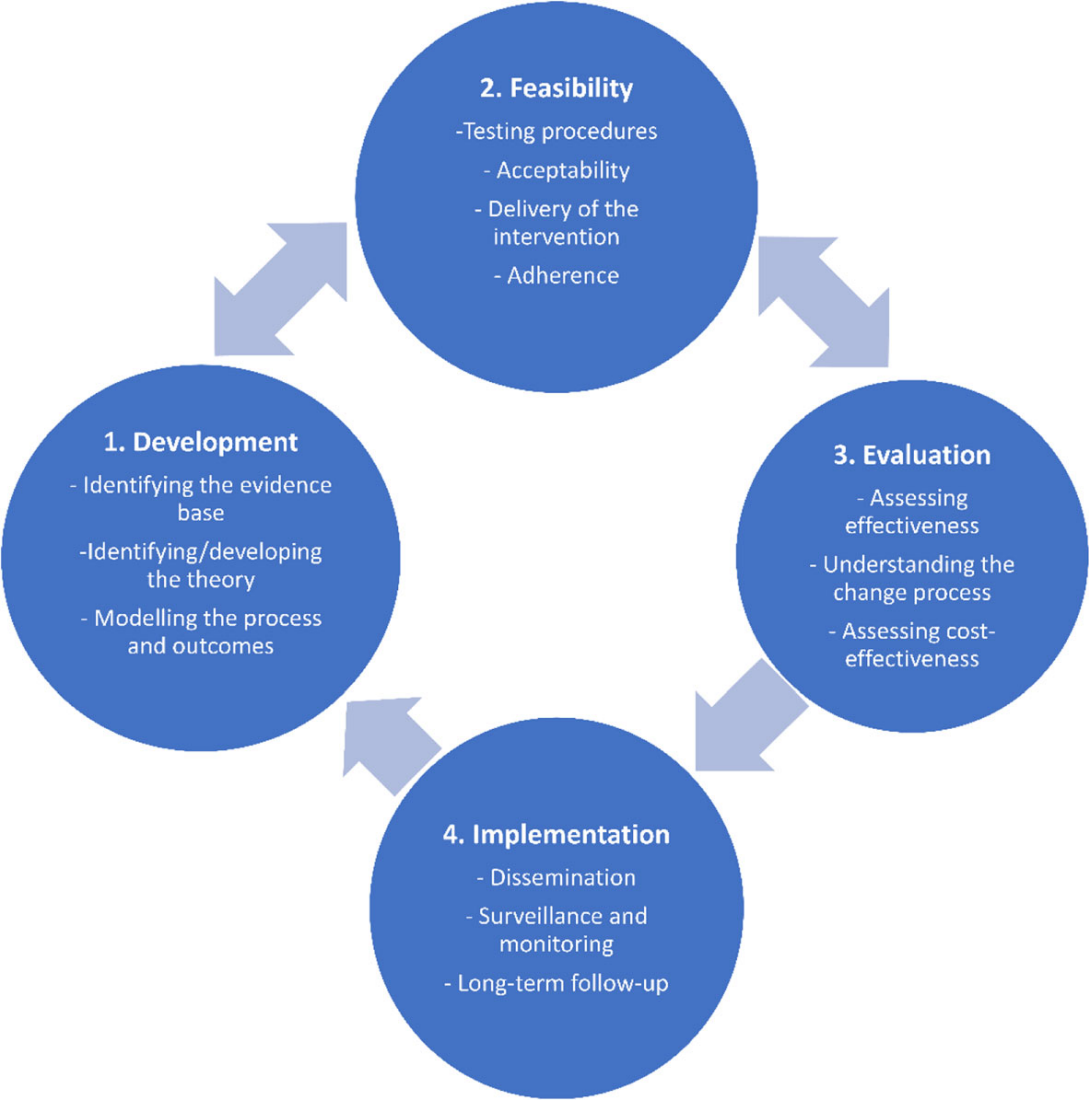
An adaptation of the MRC framework for implementation and evaluation of complex interventions

This intervention is considered complex due to the number of interacting components, number of behaviours being targeted, range of possible outcomes and the need to adapt implementation to the local settings—which has implications for evaluation, especially in assessing fidelity. Guided by the Medical Research Council Framework for designing and evaluating complex interventions [61], we present a plan for evaluation and implementation of the intervention (see Fig. 5).

We propose to select four hospitals as learning sites/case studies to test feasibility and acceptability of the intervention. The hospitals will be selected from counties that have different TB case notification rates (high vs low), in which we are able to collect reliable estimates of the outcomes of interest (see Fig. 4). We propose to choose hospitals from the Clinical Information Network where we started the preliminary work, as they have already shown readiness and willingness to improve care for children with TB and have reliable medical records. All the hospitals will undergo a sensitisation to the project and a process of getting champions to emerge with a strategy to further support them including leadership training. All will also receive the redesigned child TB training, followed by regular audits of performance in the care given to children with possible TB. Two hospitals will receive feedback with supervision by the hospital TB champion and the other two will receive feedback with supervision by outreach from TB programme officers. This will test the feasibility of these two strategies with qualitative determination of differences in preference for supervisors.

Mechanisms for delivery of feedback, i.e. how frequently, to groups or individuals, written or verbal feedback, will be allowed to adapt to each site, guided by the champions/supervisors, with each team deciding how they will go about problem solving, frequency of meetings, what goals to set for improvement etc. The data for feedback will however be standard, reporting on similar variables for the quality of care given. Workflow restructuring will be site dependent, and will evolve from the group problem-solving efforts. External support and mentorship will be available from the TB programme and the research team, who will be responsible for documenting the implementation process. The intervention will initially be delivered over 6 months in all the participating hospitals, to learn what aspects of the intervention work as intended, what are the resource costs, are the processes acceptable, practical whilst causing minimal disruption. Aspects that need refinement will go back to the development stage, and those that are effective will be adopted for implementation, learning and refining iteratively over an 18-month period.

After feasibility has been established, the evaluation will be done to establish the effectiveness of the intervention, understand the change process and assess cost-effectiveness. Simultaneous quasi-experimental interrupted time-series studies will be conducted with data prospectively collected from medical records of all paediatric admissions in the selected hospitals. Quantitative data outcomes as outlined in the logic model (Fig. 4) will include proportion of paediatric admissions including pneumonia cases with suggestive signs of TB who get correctly evaluated for TB; number of TB tests done and results; proportion of patients who get a documented differential diagnosis of TB; proportion who get started on treatment and time spent from diagnosis to treatment. Whilst a cluster randomised control trial would have been a more robust approach, this interrupted time series design is chosen for feasibility and to enable learning and refining of the intervention with local adaptations. Conduct of parallel studies in two sets of case study hospitals powered for effect will explore replication and provide effect estimates for interventions that share major components but differ in supervision, feedback and activities prioritised for problem solving. Consistent results will increase plausibility that effects are attributable to the intervention.

The quasi-experimental design will be strengthened by qualitative work which will explore the intervention process, the pathway to effect, validity of the pre-specified theory whilst describing the modifying effect of differences in context. We will collect data on the health workers’ experiences, their confidence levels, their beliefs about capabilities, decision processes etc., as guided by the logic model, to assess how well the BCW intervention functions explain what works about the intervention. For process evaluation, we will document the quality of delivery of the intervention at each site and any variabilities, assess how well the champions take up their roles, frequency of feedback and group problem solving, goals set and how all these contribute to the desired outcomes of interest, and whether there are any unintended disruptions to other clinical services. We will be looking to identify how well the starting theory explains the causal mechanisms of the outcomes, and whether other contextual factors can explain variation at the case study sites.

We also propose to also carry out an economic evaluation that will be of great use to policymakers when planning for scale up. We will document the time and effort as well as material resources used to deliver the intervention, compared to status quo. We propose to use participant observation by the champions and TB programme supervisors, and non-participant observation by the research team, all of whom will be documenting their reflections in diaries. For analysis, we will use the Theoretical Domains Framework to assess theoretical fidelity (to what extent the intervention was delivered in tandem with the intervention theory). We will also borrow from realist philosophies [62], to learn and document: “what works for whom, in what respects, in what contexts and how?” This will be important for predicting the outcomes and translating and adapting interventions for other contexts.

## Discussion

We set out to describe the process we undertook to design a contextually appropriate and theory-informed intervention to improve case detection of TB in children in Kenyan hospitals, guided by the Behaviour Change Wheel [27] and using standard intervention taxonomies as recommended by Expert Recommendations for Implementing Change (ERIC) project [20]. The behaviour change interventions identified included (1) training; (ii) modelling; (iii) persuasion; (iv) environmental restructuring; and (v) education; with the following policy categories—guidelines, regulation and communication/marketing. The process thus resulted in a multi-faceted intervention package composed of (i) redesigning of child TB training; (ii) careful selection of champions; (iii) use of audit and feedback linked to group problem solving and (iv) workflow restructuring with role specification. The intervention components were selected for their effectiveness (from literature), affordability, acceptability and practicability and designed so that NTP officers and hospital managers can be supported to implement them with relative ease, alongside their daily duties. We also provide for how the proposed intervention package can be implemented and evaluated, guided by the MRC framework for complex interventions and the Theoretical Domains Framework/Behaviour Change Wheel.

There are several implementation frameworks in literature, including those by Sheikh et al. [63], Greenhalgh et al. [64], Murray et al. [65], Damschroder et al. [66] amongst others. They all have concepts demonstrating connections between the individual and the context (organisation and wider environment, inner vs outer settings). Choice of framework often needs trade-offs between being complex enough to represent reality whilst being simple enough to be useful for policymaking, planning and research. The Behaviour Change Wheel served the purpose of providing an intuitive approach to designing an intervention to improve case detection of tuberculosis and use of TB diagnostic tests in children that seems relevant to county hospital settings in Kenya.

This approach has various strengths including the use of local empiric data to ensure the intervention is designed for the context; using consistent implementation terminologies and use of theory to describe intervention components and explain how they are intended to achieve their effects. The process further provides the opportunity to evaluate intervention delivery and effects linked to a logic model/conceptual framework. The merits of combining the BCW and ERIC taxonomy is that it enhances understanding and generalisability of the study findings. The intervention design process considered perspectives of individual health workers and the institutions expected to deliver the intervention over the long-term and is based on a well-developed understanding of existing problems from an insider perspective, which increases chances of intervention success [67]. A major assumption is that all the other structures and processes in the health system consistently function well and are in support of the proposed intervention, e.g. resources need to be consistently available, staff should be sufficient and the environment in the hospitals, community and policy space should be conducive for the intervention to work well. The major limitation is that we are yet to pilot test the intervention, and the next steps will include implanting and evaluating the process.

## Conclusion

We have designed a contextually appropriate theory-driven intervention to help address gaps in case detection of tuberculosis in children in Kenya. The intervention components were selected for their effectiveness (from literature), affordability, acceptability and practicability and designed so that TB programme officers and hospital managers can be supported to implement them with relative ease, alongside their daily duties. This work is relevant to policy and practice because it calls for a reevaluation of the strategies adopted by the existing NTP especially its approach to identifying children with TB. There is a need to review the approach to training in terms of its goals, content, pedagogy and participants with a suggestion that training should be conducted at hospitals themselves. Other practice implications include using champions and establishing social norms like teamwork and mentorship, as well as group problem solving for quality improvement and to restructure workflows in the hospitals. This work contributes to the field of implementation science by utilising clear definitions (from ERIC) and descriptions of underlying mechanisms of interventions (from the BCW) that will guide others to do likewise in their settings for similar problems.

## Supplementary Information

**Additional file 1.** Mixed Methods Conceptual Framework.

**Additional file 2.** Behavioural Analysis.

**Additional file 3.** Identifying what behaviour needs to change linked to COM-B.

**Additional file 4.** Using the TDF to expand on COM-B components identified in the behavioural diagnosis.

**Additional file 5.** Standards for Reporting Implementation Studies: the StaRI checklist.

## Data Availability

The datasets (interview transcripts and observation notes) generated and analysed for the qualitative study published elsewhere [31] are not publicly available due to maintaining confidentiality of the study participants but are available from the corresponding author on reasonable request. The summary data and underlying findings for the quantitative studies are freely available in the published papers and their supporting information files [6, 11]. Access to raw data may require additional approval from the Ministry of Health, Kenya. Requests can be facilitated by contacting the KEMRI-Wellcome Trust Research Programme’s Data Governance Committee through this email: dgc@kemri-wellcome.org.

## Abbreviations

BCW: Behaviour Change Wheel
COM-B: *Capability, Opportunity* and *Motivation* Behavioural model
DST: Drug susceptibility testing
ERIC: Expert Recommendations for Implementing Change
IPT: Isoniazid preventive therapy
NTP: National TB Programme
QUAL: Qualitative data
QUAN: Quantitative data
RTI: Respiratory tract infection
S/S: Signs and symptoms
TDF: Theoretical Domains Framework
WHO: World Health Organisation

## Footnotes

1 APEASE criteria A—Affordability P—Practicability (can be delivered as designed through the means intended to target population) E—Effectiveness and cost-effectiveness A—Acceptability (judged to be appropriate by relevant stakeholders) S—Side-effects/safety-minimal unintended consequences E—Equity (reduces or increases disparities in standard of living or well-being)
