# Supplementary material for: Improving case detection of tuberculosis in hospitalised Kenyan children—employing the behaviour change wheel to aid intervention design and implementation
Source: Implement Sci. 2020 Nov 25;15:102. doi: 10.1186/s13012-020-01061-4 (PMC7687703; doi:10.1186/s13012-020-01061-4)
Supplement: Supplementary file 2 — Additional file 2. Behavioural Analysis. [file 13012_2020_1061_MOESM2_ESM.docx]

Intervention Aim: Improving TB case detection and use of TB diagnostic tests in children in Kenya

**Behavioural target (what needs to be done and by whom?)**

1. Better clinical evaluation of possible TB patients by health workers in hospitals

- Better documentation of history and physical signs and symptoms suggestive of TB (evidenced by ticked boxes in the PAR)

2. Better use of TB diagnostic tests by health workers in hospitals

- -Better documentation of:
- -Test ordered (CXR, Mantoux, Xpert, culture), date done
- -Samples collected, and when
- -Results documented (plus date)

3. Increase in documentation of TB as a primary or secondary differential diagnosis, from the current 2.9% to double by health workers in hospitals

4. Quality improvement meetings: reflection and goal setting to improve paediatric TB care by health workers in hospitals

- Role of champion
- External supervision
- Teamwork, norms, best practices
- Improve patient flows/processes; documentation

**Possible other linked behaviours**

- Ensuring availability of paediatric admission record forms
- Ensuring availability of reagents, cartridges, specimen bottles, safety masks
- Ensuring availability of guidelines/job aides
- Designating a safe space for specimen collection
- Ensuring samples get to the lab on time
- Ensuring results get back to each patients’ file and gets reviewed by clinician
- Providing personal protective equipment
- Increasing number of HCWs trained in child TB (specimen collection, interpreting CXRs)
- Making data available for audit and feedback, and to track improvement

| **Behavioural analysis: Defining the gaps in behavioural terms** | | | | | | |
| --- | --- | --- | --- | --- | --- | --- |
| **What behaviour** | **Where & when does the behaviour occur** | **Who is involved in performing the behaviour** | **Likely impact of the behaviour if changed** | **How easy it is to change** | **Likely spill-over effect** | **Ease of measurement** |
| Better documentation of history and physical signs and symptoms suggestive of TB | Out-patient departments and hospital wards  Each visit | Clinicians,  Doctors,  Nurses (triage) | Very promising, better documentation enables audit | Promising, needs reinforcing and availability of resources | Very promising, if everyone does it | Promising, review of documents |
| Better documentation of Tests ordered (CXR, Mantoux, Xpert, culture), date done | Out-patient departments and hospital wards  Each visit | Clinicians,  Doctors | Very promising, better documentation enables audit | Promising, needs reinforcing and availability of resources | Very promising, if everyone does it | Promising, review of documents |
| Encourage better documentation of samples collected, and when (NPA, GA, IS) | Out-patient departments and hospital wards  Each visit | Clinicians,  Doctors | Very promising, better documentation enables audit | Promising, needs reinforcing and availability of resources | Promising | Promising, review of documents |
| Encourage better documentation of TB test results, date positive or negative | Out-patient departments and hospital wards  Each visit | Clinicians,  Doctors,  Lab staff  Radiology staff | Very promising, better documentation enables audit | Promising, needs motivation to follow results & availability of resources | Promising | Promising, review of documents |
| Encourage better documentation of TB as a primary or secondary differential diagnosis, from the current 2.9% to double? | Out-patient departments and hospital wards  Each visit | Clinicians,  Doctors | Very promising, better documentation enables audit | Promising, needs reinforcing | Very promising, if everyone does it | Promising, review of documents |
| Ensuring availability of paediatric admission record forms/structured forms | Out-patient departments and hospital wards  Each visit | Ward-in-charge  Administration | Very promising, it will improve documentation | Promising, needs to be prioritised: policy, hospital supplies | Promising | Promising |
| Ensuring availability of reagents, cartridges, specimen bottles, safety masks | Out-patient departments, hospital wards, (? Labs)  Each visit | Lab in charge  Administration  Ministry of Health | Very promising, it will ensure people can do tests when needed | Promising, needs Promising will to be prioritised | Promising | Promising |
| **What behaviour** | **Where does the behaviour occur** | **Who is involved in performing the behaviour** | **Likely impact of the behaviour if changed** | **How easy it is to change** | **Likely spill-over effect** | **Ease of measurement** |
| Ensuring availability of guidelines/job aides | Out-patient departments, hospital wards, (? Labs)  Each visit | Administration  Ministry of Health | Very promising, it will reinforce Promising practice | Promising, the guidelines as they stand are vague. May need revision | Promising | Promising |
| Designating a safe space for specimen collection | Out-patient departments, hospital wards, (? Labs)  Each visit | Administration | Promising but people still need to want to do it | Promising-hard  Might need restructuring, consider cost | Unpromising but worth considering-Promising | Promising |
| Ensuring samples get to the lab on time | Out-patient departments, hospital wards  Each visit | Clinicians,  Doctors,  Nurses | Promising, Promising for sample quality | Promising, requires reinforcing | Promising | Unpromising but worth considering. Needs time to be documented |
| Ensuring results get back to each patients’ file and gets reviewed by clinician | Labs, radiology,  Hospital wards,  Outpatient departments  Each visit | Clinicians,  Doctors,  Nurses  Lab & radiology | Promising, will allow audit | Promising, requires reinforcing | Promising | Promising |
| Providing personal protective equipment and encouraging consistent use | Labs, radiology,  Hospital wards,  Outpatient departments  Each visit | Administration,  Ministry of Health | Promising, people still need to use them | Promising-hard, cost implication | Promising | Promising |
| Training HCWs in child TB (specimen collection, interpreting CXRs) | Hospitals  Ministry of Health  (when to be decided by NTP) | Administration,  Ministry of Health | Very promising if made practical & done for the right staff | Promising-hard, cost implication | Very promising | Promising |
| Making data available for audit and feedback, and to track improvement | Hospitals  Ministry of Health  ? Monthly or every two months or quarterly | Health records staff  Ministry of Health | Very promising, allows for reflection and action planning | Easy, just need to pick indicators to track | Very promising | Promising |
| **What behaviour** | **Where does the behaviour occur** | **Who is involved in performing the behaviour** | **Likely impact of the behaviour if changed** | **How easy it is to change** | **Likely spill-over effect** | **Ease of measurement** |
| Providing clinical leadership, mentorship and supervision | Hospitals  Ministry of Health  Monthly or every two months or quarterly | Administration,  Ministry of Health | Very promising | Promising, needs the right people | Very promising | Promising |
| Building teamwork to ensure best practices | Hospitals  Daily | Staff and admin | Very promising | Promising, before team cohesion builds | Very promising | Promising |
| Reorganising patient flow and processes | Hospitals  Every QI cycle | Staff & admin | Very promising | Promising, needs work to ID bottlenecks | Very promising | Promising |
