# Supplementary material for: Improving case detection of tuberculosis in hospitalised Kenyan children—employing the behaviour change wheel to aid intervention design and implementation
Source: Implement Sci. 2020 Nov 25;15:102. doi: 10.1186/s13012-020-01061-4 (PMC7687703; doi:10.1186/s13012-020-01061-4)
Supplement: Supplementary file 3 — Additional file 3. Identifying what behaviour needs to change linked to COM-B. [file 13012_2020_1061_MOESM3_ESM.docx]

| **What behaviour** | **What needs to change in terms of COM-B, in order for hospital staff to improve case detection of TB and use of TB diagnostic tests in children** | | | | | |
| --- | --- | --- | --- | --- | --- | --- |
|  | **Physical capability** | **Psychological capability** | **Physical opportunity** | **Social opportunity** | **Reflective motivation** | **Automatic motivation** |
| Encourage better documentation of history and physical signs and symptoms suggestive of TB | Physical skill of history taking and physical exam | Know the correct patients to triage for TB screening | Have PAR forms  Have time to thoroughly clerk and examine | Be part of a team keen on TB detection  Have good clinical leads who mentor and emphasise good care | Hold beliefs that it is possible to Dx TB in children  Believe that diagnosing TB in children will make a difference | Have established routines and habit of considering TB as a DDx in sick children |
| Encourage better documentation of Tests ordered (CXR, Mantoux, Xpert, culture), date done | Physical skill of documenting requests & results | Know who & when to investigate | Have space for requests and results in structured forms | A culture of documenting requests and results | Hold beliefs that it is important to | Have established routines and habits of good documentation |
| Encourage better documentation of samples collected, and when (NPA, GA, IS) and tests results | Physical skill of correctly collecting appropriate specimen and documenting | Know which specimen collection method to choose and when | Availability of resources and dedicated space and time for specimen collection | Be part of a team keen on TB detection  Have good clinical leads who mentor and emphasise good care for children | Hold beliefs that it is important to | Have established routines and habits of good documentation |
| Ensuring availability of paediatric admission record forms/structured forms | Physical skill of ordering PAR forms before they stock out | Know when and how many forms to order | Have funds to regularly print PAR forms | Be part of a team keen on good documentation.  Have clinical leads who mentor and emphasise good care for children | Hold beliefs that it is important to document | Have established routines and habits of good documentation |
| **What behaviour** | **What needs to change in terms of COM-B, in order for hospital staff to improve case detection of TB and use of TB diagnostic tests in children** | | | | | |
|  | **Physical capability** | **Psychological capability** | **Physical opportunity** | **Social opportunity** | **Reflective motivation** | **Automatic motivation** |
| Ensuring availability of guidelines/job aides | Physical skill of ordering resources before they stock out | Know when and how to order resources | Have funds to ensure availability job aides | Have clinical leads who ensure consistent availability of resources | Hold beliefs that it is important to ensure availability of resources | Have established routines and habits of good stock keeping |
| Ensuring samples get to the lab on time | Physical ability to get samples to lab as soon as collected or safely store | Know when specimen should get to lab and how to appropriate store | Ensuring there’s a mechanism for prompt transport to lab and appropriate conditions for storage | Be part of a team that is comfortable in specimen transport & storage | Hold beliefs that it is important to ensure appropriate transport & storage of specimen | Have established routines and habits of specimen transport and storage |
| Ensuring results get back to each patients’ file and gets reviewed by clinician | Physical skill of correctly documenting results in patient files | Know when results should be of concern | Ensuring there’s a mechanism for prompt transfer of results to patient files and reviewed by clinicians | Be part of a team that routinely reviews patient results | Hold beliefs that it is important to document and review results | Have established routines and habits of review of results |
| Providing personal protective equipment and encouraging consistent use | Physical ability to ensure use of PPE | Know when and how PPE should be used | Resources available to buy PPE | Culture of using PPE | Holds beliefs that it is important to use PPE | Have routines and habits of using PPE |
| On-job training HCWs in child TB (specimen collection, interpreting CXRs) | Physical ability to provide on-job training for child TB | Know how and why to provide OJT | Resources available to provide OJT (time, space, equipment, skilled staff) | Culture of knowledge sharing | Holds beliefs that it is important to share knowledge | Have routines and habits of knowledge sharing |
| **What behaviour** | **What needs to change in terms of COM-B, in order for hospital staff to improve case detection of TB and use of TB diagnostic tests in children** | | | | | |
|  | **Physical capability** | **Psychological capability** | **Physical opportunity** | **Social opportunity** | **Reflective motivation** | **Automatic motivation** |
| Providing clinical leadership, mentorship and supervision | Physical ability to provide clinical leadership and mentorship | Know how and when to provide leadership and mentorship | Have dedicated processes and people to provide clinical leadership | Culture of supportive supervision | Hold beliefs that it is important to have good clinical leadership and mentorship | Have routines and habits of clinical leadership and mentorship |
| Building teamwork to ensure best practices | Physical ability to organise staff into clinical teams | Know how to use teamwork to ensure best practices | Have structures in place to ensure good teamwork like communication, handing over etc | Culture of working together as a team | Hold beliefs that it is important to work as a team | Have routines and habits of working together as a team |
| Reorganising patient flow and processes | Physical ability to reengineer patient flow | Know how and why and when to reorganise patient flows | Have space to be able to reorganise patient flows | Culture of quality improvement | Hold beliefs that it is important to reduce bottlenecks and redundancies in the system | Have routines and habits of improvement |
