# Supplementary material for: Improving case detection of tuberculosis in hospitalised Kenyan children—employing the behaviour change wheel to aid intervention design and implementation
Source: Implement Sci. 2020 Nov 25;15:102. doi: 10.1186/s13012-020-01061-4 (PMC7687703; doi:10.1186/s13012-020-01061-4)
Supplement: Supplementary file 4 — Additional file 4. Using the TDF to expand on COM-B components identified in the behavioural diagnosis. [file 13012_2020_1061_MOESM4_ESM.docx]

| COM-B | TDF linked to COM-B | Relevance of the domain |
| --- | --- | --- |
| Psychological capability | Knowledge | Awareness of steps in diagnosing TB in children; of the available diagnostic tests. Rationale. Do they know what they should do and when and why? |
|  | Cognitive & interpersonal skills | Proficiency in diagnosing TB; using diagnostic tests. Acquired through practice |
|  | Memory attention and decision processes | Ability to retain information, to consistently remember to order TB tests to do, and when |
|  | Behavioural regulation | Self-monitoring; how to break a habit e.g. missed diagnosis. Anything in place to prompt them to make a diagnosis and to monitor whether or not they have |
| Physical capability | Physical skills | Are they physically able/proficient in diagnosing TB; collecting specimen; using diagnostic tests. Acquired through practice |
| Social opportunity | Social influences | Social norms/group conformity to good clinical practices; group identity; modelling/role models.  How might the views/opinions of colleagues/patients influence their decision to diagnose TB in children |
| Physical opportunity | Environmental context & resources | Organisational processes and patient flows; resources like job aides, PPE, reagents. Aspects of the environment that influence whether or not they diagnose TB in children |
| Reflective motivation | Social/professional role & identity | Do they think it is part of their job e.g. to collect specimen (seniors struggled with this) |
|  | Beliefs about capability | Are they confident diagnosing TB in children; collecting specimen? How difficult or easy? |
|  | Optimism | Do they think it’s something that can be done? How confident are they of this? |
|  | Intentions | Have they made a decision (not) to do it? |
|  | Goals | How much do they want to do it? |
|  | Beliefs about consequences | DO they believe doing it or not makes a difference? |
| Automatic motivation | Reinforcement | Anything to motivate or demotivate them? |
|  | Emotion | Does it evoke an emotional response e.g. uncomfortable when babies cry during specimen collection; fear of reprimand from rich relatives |
