# Supplementary material for: Improving case detection of tuberculosis in hospitalised Kenyan children—employing the behaviour change wheel to aid intervention design and implementation
Source: Implement Sci. 2020 Nov 25;15:102. doi: 10.1186/s13012-020-01061-4 (PMC7687703; doi:10.1186/s13012-020-01061-4)
Supplement: Supplementary file 5 [file 13012_2020_1061_MOESM1_ESM.docx]

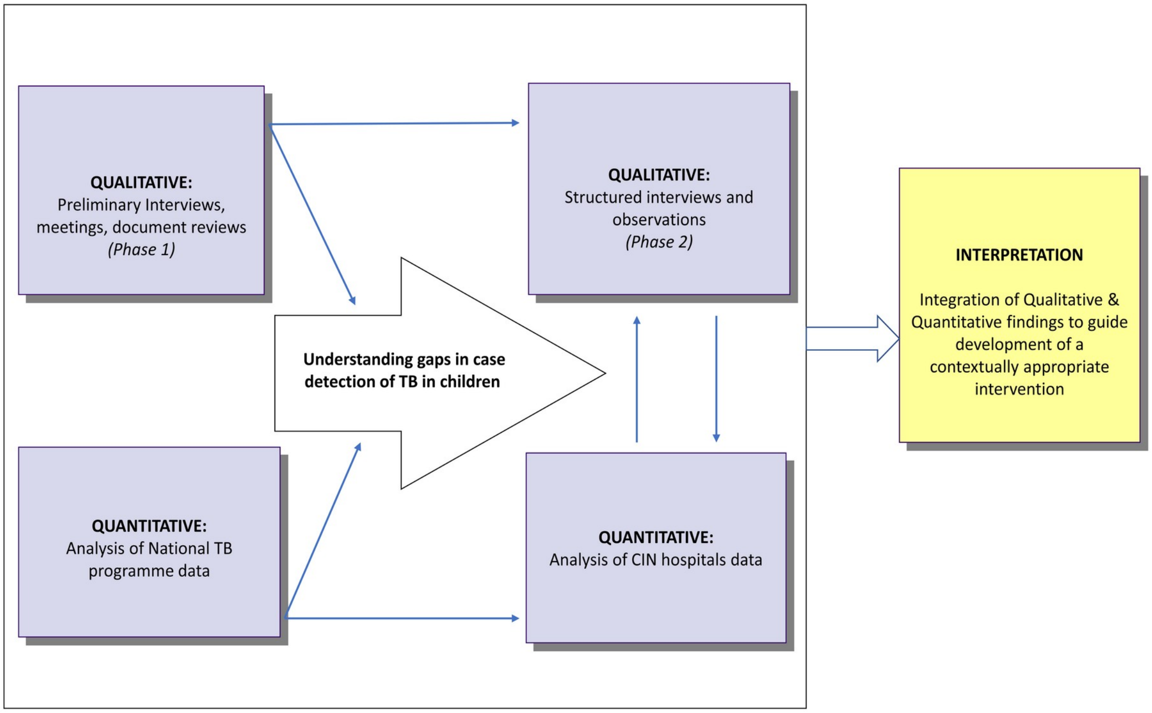
QUALITATIVE:
Preliminary Interviews, meetings, document reviews
(Phase 1)
QUALITATIVE:
Structured interviews and observations
(Phase 2)
Understanding gaps in case detection of TB in children
QUANTITATIVE:
Analysis of National TB programme data
QUANTITATIVE:
Analysis of CIN hospitals data
INTERPRETATION
Integration of Qualitative & Quantitative findings to guide development of a contextually appropriate intervention
